# Reverse shoulder arthroplasty versus hemiarthroplasty versus non-surgical treatment for older adults with acute 3- or 4-part fractures of the proximal humerus: study protocol for a randomised controlled trial (PROFHER-2: PROximal Fracture of Humerus Evaluation by Randomisation – Trial Number 2)

**DOI:** 10.1186/s13063-023-07259-3

**Published:** 2023-04-13

**Authors:** Amar Rangan, Stephen Gwilym, Ada Keding, Belen Corbacho, Lucksy Kottam, Catherine Arundel, Elizabeth Coleman, Livio DiMascio, Catherine Hewitt, Valerie Jones, Jamila Kassam, Catriona McDaid, Natasha Mitchell, Andrew Mott, Grace O’Carroll, Puvan Tharmanathan, David Torgerson

**Affiliations:** 1 Department of Health Sciences & HYMS, University of York, York, YO10 5DD UK; 2 NDORMS, University of Oxford, Headington, Oxford, OX3 7LD UK; 3 York Trials Unit, Department of Health Sciences, University of York, York, YO10 5DD UK; 4 Astellas Pharma S.A.,Torre Emperador Castellana, Paseo de La Castellana- nº 259, D - Planta 31, Madrid, 28046 Spain; 5 South Tees Hospitals NHS Trust, Marton Road, Middlesbrough, TS4 3BW UK; 6 Barts Health NHS Trust, The Royal London Hospital, Whitechapel Road, London, E1 1FR UK; 7 Northern General Hospital, Herries Road, Sheffield, S5 7AU UK; 8 Queen Mary University of London, Whitechapel, London, E1 2AD UK

**Keywords:** Proximal humeral fracture, Reverse shoulder arthroplasty, Hemiarthroplasty, Non-surgical treatment, Randomised trial

## Abstract

**Background:**

Proximal humerus fractures (PHF) are common and painful injuries, with the majority resulting from falls from a standing height. As with other fragility fractures, its age-specific incidence is increasing. Surgical treatment with hemiarthroplasty (HA) and reverse shoulder arthroplasty (RSA) have been increasingly used for displaced 3- and 4-part fractures despite a lack of good quality evidence as to whether one type of arthroplasty is superior to the other, and whether surgery is better than non-surgical management. The PROFHER-2 trial has been designed as a pragmatic, multicentre randomised trial to compare the clinical and cost-effectiveness of RSA vs HA vs Non-Surgical (NS) treatment in patients with 3- and 4-part PHF.

**Methods:**

Adults over 65 years of age presenting with acute radiographically confirmed 3- or 4-part fractures, with or without associated glenohumeral joint dislocation, who consent for trial participation will be recruited from around 40 National Health Service (NHS) Hospitals in the UK. Patients with polytrauma, open fractures, presence of axillary nerve palsy, pathological (other than osteoporotic) fractures, and those who are unable to adhere to trial procedures will be excluded. We will aim to recruit 380 participants (152 RSA, 152 HA, 76 NS) using 2:2:1 (HA:RSA:NS) randomisation for 3- or 4-part fractures without joint dislocation, and 1:1 (HA:RSA) randomisation for 3- or 4-part fracture dislocations. The primary outcome is the Oxford Shoulder Score at 24 months. Secondary outcomes include quality of life (EQ-5D-5L), pain, range of shoulder motion, fracture healing and implant position on X-rays, further procedures, and complications. Independent Trial Steering Committee and Data Monitoring Committee will oversee the trial conduct, including the reporting of adverse events and harms.

**Discussion:**

The PROFHER-2 trial is designed to provide a robust answer to guide the treatment of patients aged 65 years or over who sustain 3- and 4-part proximal humeral fractures. The pragmatic design and recruitment from around 40 UK NHS hospitals will ensure immediate applicability and generalisability of the trial findings. The full trial results will be made available in a relevant open-access peer-reviewed journal.

**Trial registration:**

ISRCTN76296703. Prospectively registered on 5th April 2018

**Supplementary Information:**

The online version contains supplementary material available at 10.1186/s13063-023-07259-3.

## Administrative information

**Table:** 

Title {1}	Reverse shoulder arthroplasty versus hemiarthroplasty versus non-surgical treatment for acute 3- or 4-part fractures of the proximal humerus in older adults: protocol for a randomised controlled trial (PROFHER-2)
Trial registration {2a and 2b}	ISRCTN76296703 Prospectively registered on 5th April 2018
Protocol version {3}	Version 1.3 (20/04/2022)
Funding {4}	This project was funded by the National Institute for Health Research: Health Technology Assessment. Reference: 16/73/03 The Views expressed are those of the author(s) and not necessarily those of the NIHR or the Department of Health and Social Care
Author details {5a}	SPIRIT guidance: Affiliations of protocol contributors
Name and contact information for the trial sponsor {5b}	South Tees Hospitals NHS Foundation Trust Marton Road, Middlesbrough, TS4 3BW
Role of sponsor {5c}	The study sponsor and funder have had no direct role in study design nor in the collection, management, analysis or interpretation of data. They will have no role in the writing of associated publications and the decision to submit papers for publication

## Introduction

### Background and rationale {6a}

Proximal humerus fractures (PHFs) are common and painful injuries, with the majority (about 90%) resulting from falls from a standing height [1]. Their incidence rises markedly with age, being highest in those aged 70 years and over, and are about three times more common in women than men [2]. The frequency of these fractures is expected to increase due to the growing incidence of fragility fractures secondary to an ageing population. PHFs are associated with disability and loss of independence and have a negative impact on health-related quality of life [3–5].

The pattern of injury varies, and the fracture may be undisplaced or displaced. Undisplaced fractures are effectively treated non-surgically, but displaced fractures trigger treatment uncertainties. The three key elements of displaced fractures are as follows: the number of displaced fractured segments or “parts” (two-, three- or four-part fractures); whether the joint surface itself is fractured; and whether there is an associated shoulder joint dislocation (found to be between 5% and 8.6% of PHF [6, 7]). These fractures can be treated either surgically or non-surgically. Non-surgical treatment involves resting the injured arm in a sling for around 3 weeks to allow initial healing, followed by physiotherapy. Surgical treatment generally involves internal fixation and preservation of the humeral head; replacement of the humeral head (hemiarthroplasty); or replacement of the humeral head and glenoid/socket (reverse shoulder arthroplasty).

The PROFHER trial found that surgical treatment was not superior to non-surgical treatment for the majority of adults with displaced PHF [8]. The trial aimed to recruit a population that reflected normal PHF epidemiology and therefore only a quarter of the study population had three- or four-part fractures. The findings provide unparalleled evidence for the management of the majority of displaced PHFs but the effectiveness of reverse shoulder arthroplasty (RSA) for more complex fractures (3- or 4-part) was not tested within that trial.

There are reports in the literature of case series of RSA for 3- or 4-part PHFs [9, 10], and observational studies comparing RSA against hemiarthroplasty (HA) [11, 12]. A systematic review suggests that using RSA for fracture results in reliable pain relief, functional range of movement and acceptable levels of patient satisfaction [13]. There is, however, an awareness of the potential complications of RSA, with up to 24% of patients reported as having a minor or major complication following surgery [14]. Another systematic review comparing HA with RSA for treatment of PHF found that pain and functional outcomes were similar in the two groups, but RSA was associated with 4.0 times greater odds of a postoperative complication [15]. No differences in patient-reported outcomes were noted at 12 months follow-up for RSA over non-surgical treatment other than marginally better VAS pain scores in the RSA group in a relatively small randomised trial [16]. Given the lack of good quality evidence, there is clear clinical uncertainty regarding the use of arthroplasty as a treatment for more complex (3- or 4-part) PHFs.

Despite the risk profile, the higher costs associated with RSA, and the presence of clinical uncertainty, its use is increasing over time [17, 18]. Data from the 2021 National Joint Registry report [19] confirms this trend in the UK, where the use of RSA for all indications has more than doubled over the 5 years from 2014, and RSA is being used nearly twice as often as HA for PHFs. The James Lind Alliance priority setting partnership on surgery for shoulder pain identified the use of RSA for 3- and 4-part PHFs as a key research priority [20]. There is a clear need for a sufficiently powered randomised controlled trial (RCT) investigating the effectiveness of RSA compared to HA and non-surgical treatment for 3- and 4-part PHFs.

The PROFHER-2 trial was therefore designed as a pragmatic, multicentre randomised controlled, three-arm trial to compare the clinical effectiveness and cost-effectiveness of RSA vs HA vs non-surgical (NS) treatment in patients with 3- and 4-part PHF.

### Objectives {7}

To investigate the clinical and cost-effectiveness of RSA versus HA for patients presenting with 3- and 4-part PHFs. Additionally, the effectiveness of surgery will be compared to non-surgical treatments (NS). The trial will:

i. Undertake a randomised parallel group comparison to determine if RSA is superior to HA in treating 3- and 4-part PHFs based on change in the Oxford Shoulder Score (OSS) at 24-month post-randomisation.
ii. Undertake a randomised parallel group comparison to determine if surgery is superior to NS in treating three- and four-part PHFs based on change in the OSS at 24 months.
iii. Compare secondary outcomes of pain, range of motion, and OSS at 6 and 12 months between the trial arms using appropriate analytic models; describe and compare safety data including complications and adverse events between the trial arms.
iv. Conduct a detailed economic evaluation to compare the cost-effectiveness of the comparisons described in objectives i and ii above at 24 months.
v. Investigate the association of baseline characteristics (demographics, grip strength) with poor functional outcomes at 24 months.

### Trial design {8}

PROFHER-2 is a pragmatic multi-centre, randomised controlled, three-arm superiority trial with parallel groups including a cost-effectiveness analysis. There was an internal pilot to explore the feasibility of recruitment.

## Methods: participants, interventions, and outcomes

### Study setting {9}

Participants will be recruited from approximately 40 centres (UK NHS hospitals) that regularly treat PHFs. Screening to identify patients eligible for the trial will occur in the orthopaedic trauma or fracture clinics and orthopaedic or trauma wards of participating NHS hospitals (Additional file 1).

### Eligibility criteria {10}

Patients who meet all the inclusion criteria, and none of the exclusion criteria will be eligible.

Inclusion criteria:

- Adult patients aged 65 years or over
- Radiographically confirmed acute 3-part (including surgical neck) or 4-part displaced fracture of the proximal humerus (Neer Classification) [21] including head-splitting fractures of the humeral head and fracture dislocations
- Trial interventions can be provided within 5 weeks of injury
- Patient is deemed by the clinical care team to be fit for surgery
- Able to provide full informed consent

Exclusion criteria:

- Patient unable to adhere to trial procedures or complete questionnaires
- Poly-trauma: where one or more additional fractures, which may affect the outcome measures for the trial are present
- Open fractures or fractures where there is severe soft tissue compromise requiring urgent surgery
- Pathological (other than osteoporotic) fractures
- Presence of axillary nerve palsy (given that this results in a weakening of the deltoid muscle, upon which the shoulder relies for function).

### Who will take informed consent? {26a}

Post screening, potential participants will be provided with information about the study including a patient information sheet and a short infographic outlining the possible treatment allocations. The patient information sheet will be reviewed if new information arises during the study which may affect an individual’s willingness to take part.

The potential participant will be allowed as much time as they wish to consider the information, and patients will have the opportunity to ask questions of the treating clinician and the local research team before consent for the study is obtained. Consent will be obtained by a member of the local research team.

As part of the consent process, consent will be sought for follow-up beyond the duration of the trial to allow the possibility of future long-term follow-up, which may include accessing relevant routinely collected data such as the National Joint Registry.

### Additional consent provisions for collection and use of participant data and biological specimens {26b}

Not applicable, no biological specimens will be collected within the PROFHER-2 trial.

## Interventions

### Intervention description {11a}

Both surgical interventions (RSA and HA) will be performed under general anaesthesia and anterior (delto-pectoral) or superior (McKenzie type) surgical approaches may be used as per the treating surgeon’s usual practice. Along with the risks of general anaesthesia, both surgical interventions have potential risks and complications.

#### Reverse shoulder arthroplasty (RSA)

During RSA surgery, the fractured articular head fragment of the humerus is removed. The joint is replaced with a reversed geometry prosthesis, where the glenoid surface (socket) receives a prosthetic hemisphere, and the humeral side receives a prosthesis that usually has a stem that anchors into the humerus and bears a socket at the top. The implant design aims to alter the joint biomechanics, making the deltoid muscle more efficient at moving the shoulder without reliance on the rotator cuff muscles.

#### Hemiarthroplasty (HA)

During HA surgery, the fractured articular head fragment of the humerus is removed. The humerus is then prepared to accept a humeral stem implant that bears a spherical head at the top as a replacement for the fractured humeral head. The remaining tuberosity fragments and associated rotator cuff are repaired to the proximal humerus and prosthesis, to reconstruct “normal” anatomy around the prosthesis. The native glenoid is not instrumented and articulates with the replaced humeral head component, thus only half the joint is replaced in this procedure.

#### Non-surgical treatment (NS)

Non-surgical treatment involves supporting the injured arm in a sling for a period of 3 weeks for comfort as in the PROFHER trial [8] and participants are provided with a sling care advice leaflet at the time of randomisation. The arm and shoulder are then gently mobilised under supervision of a physiotherapist with the aim of increasing the range of motion and performing active exercises as tolerated. Physiotherapy sessions are tailored and include advice and education on a home exercise programme predominantly based on daily functional tasks. The physiotherapy sessions include a combination of exercise, soft tissue techniques, joint mobilisations, stretching and relaxation techniques. The physiotherapy pathway for non-surgical treatment follows what was used successfully in the PROFHER trial [8]. As 3- and 4-part fractures will be included in this trial, we have allowed for a median of 12 physiotherapy sessions being required (compared to eight required in PROFHER). The exact treatments may be individualised to ensure that rehabilitation is tailored to individual needs in line with routine conservative care.

Non-surgical treatment has the advantage of avoiding the risks of anaesthesia and surgery. If pain is persistent or function remains poor after non-surgical treatment, delayed surgery may be performed at clinical discretion. This would not usually be considered before 6 months to allow an adequate period of rehabilitation to be pursued.

### Explanation for the choice of comparators {6b}

The need for comparison of surgical options for this patient group was part of the funder commission (NIHR 16/73). HA was the most commonly used surgical option at the point RSA was introduced in practice and was thereby chosen as the surgical comparator. Acknowledging the results from the PROFHER trial which found no significant difference between surgical and non-surgical treatments, it was deemed necessary to also include a non-surgical comparator to determine the best method for treating these fractures.

### Criteria for discontinuing or modifying allocated interventions {11b}

PROFHER-2 is a pragmatic trial comparing established interventions; thus, there will be no formal criteria for the discontinuation or modification of allocated interventions. Any modifications or changes to allocated interventions or any further surgical treatment will be recorded and reported in the trial documentation.

### Strategies to improve adherence to interventions {11c}

This study does not include any specific strategies to improve intervention adherence given the pragmatic nature of the trial and the requirement to follow local policies on surgical and non-surgical treatment.

### Relevant concomitant care permitted or prohibited during the trial {11d}

Following surgery (RSA or HA) a supportive arm sling is provided for comfort and a graduated rehabilitation programme followed. Physiotherapy guidance for RSA and HA developed through consensus by the British Elbow and Shoulder Society physiotherapists for the purposes of this trial will be provided to all trial centres. The guidance recommends supervised physiotherapy with the aim of gradually increasing range of motion and function. Due to the biomechanics of RSA and the increased risk of dislocation [22], internal rotation (i.e., hand behind back movement) is avoided following RSA to protect the joint until clinician review (at around 6 weeks).

Perioperative care provided to participants will be recorded; however, there is no standardisation of perioperative care, in line with the pragmatic nature of PROFHER-2. Perioperative care is defined as the period from start of anaesthesia to the discharge of the patient from the ward following surgery.

Intravenous antibiotics may be given prophylactically to minimise the risk of subsequent prosthetic infection. The type of analgesia (regional or intravenous) and antibiotic use will be recorded within the case report form.

### Provisions for post-trial care {30}

Following completion of the trial, participants will return to the care of their treating healthcare professional to determine if any further treatment is required.

### Outcomes {12}

The primary outcome is the OSS at 24 months. The OSS provides a total score based on the person’s subjective assessment of pain and activities of daily living, has established content validity in post-operative patients [23], and has been used successfully in other large surgical trials of shoulder disorders [8, 24–26]. This outcome measure has also been chosen to allow comparison with the data obtained from the PROFHER trial [8].

Secondary outcomes will include:

1. Quality of life using EQ-5D-5L [27]
2. Pain using the Patient-Reported Outcomes Measurement Information System (PROMIS) pain interference measure [28]
3. Pain using a visual analogue pain scale (from no pain to worst imaginable pain – using a 10 cm line)
4. Range of shoulder motion (recorded at discharge from physiotherapy and independently assessed at 6 months post-randomisation i.e., not by the treating surgeon)
5. Healing and implant position using AP and axillary (and scapular Y view if available) X-rays taken at 6 months post-surgery
6. Further procedures and complications.

In addition, grip strength on the uninjured side will be collected at baseline using a Jamar Dynamometer [29]. This will be used to assess frailty and as a predictor of morbidity and mortality. Physiotherapy utilisation will also be collected during the trial using case report forms specifically developed for the study.

### Participant timeline {13}

The participant flowchart can be found in Appendix 1.

### Sample size {14}

The minimum clinically important difference (MCID) for the OSS has been established for this patient population at five points [8, 30, 31]. As RSA is more extensive and expensive, it would be essential for RSA to be superior to HA by at least five OSS points to justify its use. Surgical intervention carries higher risks and costs compared to non-surgical treatment and therefore looking for a larger difference in OSS of 6 points would be appropriate to justify the use of surgical treatment over non-surgical treatment {van Kampen}. Hence, a mean difference of five OSS points will be sought between the two surgical arms and six OSS points between each surgical arm and non-surgical treatment [32].

Assuming a standard deviation of 12, 90% power and 5% two-sided statistical significance, 320 participants are required to power all three group comparisons. Assuming 15% attrition over 24 months, the total recruitment target is 380 (152 RSA, 152 HA, 76 NS — figures initially using a 2:2:1 ratio (HA:RSA:NS)). Included in this sample are patients who are allocated 1:1 to one of the two surgery arms only (i.e. patients who require general anaesthetic for fracture dislocation reduction). The proportion of participants requiring general anaesthesia to reduce an associated joint dislocation was monitored as part of the pilot phase and was consistently found to be higher than anticipated (approximately 35% at the end of the pilot phase, compared to the expected 5%). As such an allocation ratio of 1:1:1 replaced the previous allocation ratio of 2:2:1 for participants randomised to all three arms (where there is no associated dislocation that needs general anaesthesia for reduction). This was implemented on 12th October 2020 to maintain power for all planned comparisons, and the initial sample size target remained unchanged.

### Recruitment {15}

All potential participants identified in the orthopaedic trauma clinics and orthopaedic trauma wards are provided with a patient information sheet by a member of the site research team, which links to a one-page infographic designed to help present the study and treatments to patients in a simplified manner. An Associate Principal Investigator (API) scheme will be utilised at participating centres to involve Specialty Trainees in Trauma and Orthopaedic Surgery to coordinate study recruitment, particularly when Research Nurses or Associates may not be available. The APIs will be trained in study processes and will be supervised by the site Principal Investigator. The study will use approximately 40 centres (NHS hospitals) that regularly treat proximal humeral fractures, to recruit on average 127 participants per year, over a 3-year recruitment period.

## Assignment of interventions: allocation

### Sequence generation {16a}

Two separate allocation sequences will be generated: one using a 1:1 ratio (RSA: HA) which will allocate patients with an associated dislocation requiring general anaesthesia for reduction, and the other using a 2:2:1 ratio (RSA:HA:NS) will allocate patients with no associated dislocation or a dislocation not requiring general anaesthesia for reduction. Sequences will be stratified by centre, using permuted blocks of varying sizes. A further sequence will be generated using a 1:1:1 (RSA:HA:NS) ratio, to replace the 2:2:1 sequence for reasons covered under {14} above.

### Concealment mechanism {16b}

A statistician at York Trials Unit (YTU) who will not be undertaking the analysis of the trial will generate the final allocation sequences for the trial using Stata v.15.

### Implementation {16c}

The research teams at sites will contact YTU, either by telephone or via the internet, to access a secure central randomisation service. The randomisation service will record information and check patient eligibility to avoid inappropriate entry of patients into the trial.

## Assignment of interventions: blinding

### Who will be blinded {17a}

As the treatments cannot be adequately concealed, it is not possible to blind clinicians or participants to their treatment allocation. Trial statisticians analysing the data will not be blinded.

### Procedure for unblinding if needed {17b}

Not applicable. Unblinding is not required.

## Data collection and management

### Plans for assessment and collection of outcomes {18a}

Data will be collected at baseline and 6, 12, and 24 months via either paper questionnaire or telephone. The data collection timetable is outlined in Appendix 2.

At baseline, the OSS and EQ-5D-5L will be collected with both an assessment of pre-injury and post-injury scores. PROMIS and pain VAS will also be collected. Epidemiological data will be collected including patient demographics, comorbidities, prior injury to the affected arm, and smoking status. Details of surgical and medical care provided will be recorded.

Data regarding any physiotherapy provided will be collected until discharge from physiotherapy. This will include the number and length of sessions, care provided, and range of motion at discharge.

In addition, at 6 months participants will have an X-ray and assessment of range of motion.

### Plans to promote participant retention and complete follow-up {18b}

Participants will receive a pen with the PROFHER-2 logo with their follow-up questionnaires at 12- and 24-month post-randomisation, as evidence suggests that this improves response rates [33].

Also, there is some evidence to suggest that contacting participants in advance of sending a questionnaire (pre-notification) may help to increase response rates; however, the evidence is not of high certainty and therefore additional studies are required [34, 35]. As a result, a study within a trial (SWAT) was embedded within this trial to test a pre-notification newsletter.

The SWAT was implemented on 29th April 2021, and all participants who were fully participating (i.e., have not fully withdrawn, withdrawn from postal follow-up or have died) and due to their 24-month follow-up after this were included in the SWAT. There were no additional inclusion or exclusion criteria. It was intended for the SWAT to run until the end of the study follow-up; however, it ended early on 30th April 2022. The decision to stop the SWAT was based on the steer from public contributors on the perceived benefit of maintaining participant engagement and to ensure a high response rate to the 24-month questionnaire with all participants in the trial and not just those randomised to receive the intervention. Participants were randomised to the SWAT using a 1:1 ratio (pre-notification newsletter and cover letter, or to receive neither). Generation of the allocation sequence was undertaken independently by a researcher not involved with the follow-up of participants. The cover letter and newsletter were sent 2–4 weeks prior to the 24-month questionnaire.

From August 2022, the pre-notification newsletter and cover letter have been merged into a single pre-notification newsletter which will be sent 4 weeks prior to the 12- and 24-month participant questionnaires. This will be for all participants recruited into the PROFHER-2 trial, and who remain as fully participating. In addition, all participants who reach their 12- and 24-month follow-ups will receive a £5 voucher at each of these timepoints along with their questionnaire, as incentives have been shown to increase retention and questionnaire responses in clinical trials [34].

### Data management {19}

Participant data will be recorded on case report forms (CRF). Separate CRFs will be used to collect clinical information and patient reported information.

To ensure high-quality data, the CRFs will be processed at YTU, using a licensed, automated, electronic system which allows data to be entered, checked and validated. A study-specific data management plan will document further details regarding the specifics of processing of this data.

Study documentation will be archived and retained in accordance with Good Research Practice and UK Law for 5 years after study completion in the Trial Master and Investigator Site Files, after which time this information will be securely destroyed.

### Confidentiality {27}

To maintain confidentiality participants will be assigned a unique four-digit number (study ID) which will be used to identify them throughout the trial. All data for the trial will be stored and handled according to relevant data protection principles and regulations.

Participant identifiable information will be stored on a secure data management system accessible only by trial staff via individual passwords. Where participant identifiable data is held in a printed format, it will be held separate from participant CRFs and kept in locked filing cabinets accessible only to the research team.

### Plans for collection, laboratory evaluation and storage of biological specimens for genetic or molecular analysis in this trial/future use {33}

Not applicable. There is no collection of biological specimens in the PROFHER-2 trial.

## Statistical methods

### Statistical methods for primary and secondary outcomes {20a}

All analyses will be undertaken using the principle of intention-to-treat, where participants are analysed as randomised, regardless of what treatment they received. Analysis will be undertaken using Stata v17 (or later), and models will be assessed at a 5% significance level, unless otherwise stated. All analyses will be preplanned and detailed in a statistical analysis plan, which will be approved by the trial oversight committees prior to analysis.

### Primary analyses

The primary outcome is the OSS at 24-month post-randomisation. The difference in OSS at 24 months will be compared between the arms of the trials using mixed effects models. The model will adjust for a dummy variable for the change in allocation ratio and relevant baseline characteristics, including pre-fracture OSS, baseline grip strength, and presence of an associated dislocation (if applicable), as well as OSS at interim follow-ups, with correlation within each participant over time modelled by an appropriate covariance structure. The treating surgeon will be included as a random effect; if clusters are too small, the centre will be included instead.

Two separate models will be used. The first model will compare the two surgical arms and include all participants randomised to either of these arms. The second model will compare the surgical and non-surgical arms but will only include participants who were eligible to receive NS as an allocation (i.e., excluding anyone with a dislocation that required general anaesthesia for reduction) and will be run once for the RSA v NS comparison, and then run separately for the HA v NS comparison. Both models will follow the principle of intention-to-treat except for a pre-specified complier average causal effect (CACE) analysis of the primary outcome.

### Secondary analyses

The secondary outcomes (pain, range of motion, estimates of OSS at 6 and 12 months) will be analysed in a similar way to the primary, using appropriate models and adjusting for the same covariates as the primary models.

### Interim analyses {21b}

There are no planned interim analyses for the trial and no formal stopping guidelines in relation to the outcomes of the trial. There was an internal pilot phase, where recruitment and retention aims were assessed in line with the feasibility of the trial — the funder approved the continuation of the trial past the pilot phase. No comparison of the primary outcome, or any other formal analysis, was undertaken as part of the pilot phase.

### Additional analyses {20b)

Sensitivity analyses will be undertaken to explore the effect of surgeon expertise in the RSA vs HA comparison. Details of the SWAT analysis will be included within the publication of the results [36].

### Methods in analysis to handle protocol non-adherence and any statistical methods to handle missing data {20c}

The primary analysis models will be repeated using data sets where the primary outcome (OSS at 24 months) and any of the model covariates that are missing have been imputed using appropriate imputation methods.

Additionally, analyses will be undertaken to explore the effect of non-compliance, where participants do not receive the treatment to which they were randomised—such as using a complier average causal effects (CACE) analysis.

#### Health economic analysis

The economic evaluation will assess the cost-effectiveness of the three competing interventions. The analysis will be conducted from the perspective of the UK National Health Services (NHS) and Personal Social Services (PSS). A health economics analysis plan (HEAP) will be written, and agreed with the oversight committees, before any analyses are undertaken. Any subsequent amendments to the plan will be clearly documented.

Self-reported questionnaires and hospital forms will be used to evaluate resource use and associated costs over the follow-up of the trial. Cost components will compromise hospital stay (initial and subsequent inpatient episodes, outpatient hospital visits and A&E hospital admissions) and primary care consultations (e.g. GP, nurse and physiotherapy). An accurate record of procedures at the hospital level (e.g. centres in the trial) will be put in place to record per patient information (e.g. surgical procedures, complications related to the surgical intervention, and other medical complications). Costs relating to surgical procedures will be based on time in theatre, staff time, consumables and devices, and nights in hospital after the procedure. These data will be collected via a form that will be specifically designed for this trial. Similarly, physiotherapy treatment logs will be completed by physiotherapists providing patient care. Cost components for health resource use will be derived from established national costing sources such as NHS Reference Costs [37], PSSRU Unit costs of health and social care [38], and the British National Formulary [39]. Unit costs will be multiplied by resource use to obtain a total cost for each patient.

The primary outcome for the economic analysis will be the additional cost per quality-adjusted life year gained (QALY). Value for money will therefore be estimated in terms of cost per QALY following an intention-to-treat approach using EQ-5D-5L data. The EQ-5D-5L will be collected at Baseline, 6 months, 12 months and 24 months’ follow-up. The overall difference in EQ-5D index scores between the two arms will be examined through regression methods, consistent with the model selected in the statistical analysis. The EQ-5D health states will be valued using the mapping function developed by van Hout et al. [40] and following the NICE position statement [41] QALYs will be calculated by plotting the utility scores at each of the three time points and estimating the area under the curve [42]. A discount rate will be applied to all costs and QALYs accrued after 12 months at a rate of 3.5% per annum in line with NICE guidance [43].

For the analysis, regression methods will be used to allow for differences in prognostic variables. Incremental cost-effectiveness ratios and net-benefit statistics will be calculated. The pattern of missing data will be analysed and handled by means of multiple imputation (MI) [44]. A range of sensitivity analyses will be conducted to test the robustness of the results under different scenarios, including probabilistic sensitivity analysis. In case of positive results of the trial, we will recommend that costs and outcomes will be extrapolated and modelled over a longer time horizon than captured by the trial (e.g. lifetime of the patient).

### Plans to give access to the full protocol, participant-level data and statistical code {31c}

This manuscript constitutes a complete representation of the trial protocol. The full protocol and related documents for the PROFHER-2 trial are available on the NIHR website [45]. Anonymised participant-level data may be requested from YTU after trial completion — the approval of any data requests is at the discretion of the Chief Investigator and YTU.

## Oversight and monitoring

### Composition of the coordinating centre and trial steering committee {5d}

#### Trial Coordination

The coordination of the PROFHER-2 trial will be managed by YTU in collaboration with the sponsor and chief investigator. The YTU team will include the trial manager, trial coordinators, data management and support staff.

#### Trial Management Group

The PROFHER-2 trial management group will include the sponsor, chief investigator, co-applicants (clinicians and other researchers), patient representatives, the trial manager, trial coordinators, statisticians, and health economists. The management group will meet every 3 months throughout the duration of the trial.

#### Trial Steering Committee

The PROFHER-2 trial steering committee (TSC) will include an independent chair, two independent clinical members with experience relevant to the trial, and a patient representative. They will meet at least once a year throughout the duration of the trial.

### Composition of the data monitoring committee, its role and reporting structure {21a}

The PROFHER-2 data monitoring committee (DMC) will be composed of members independent of the sponsor and trial team and will include an orthopaedic surgeon, a statistician, and one other clinician.

The DMC will meet at least once a year throughout the trial and will report to the TSC.

### Adverse event reporting and harms {22}

Adverse events (AEs) are defined as any untoward medical occurrence (i.e. any unfavourable and unintended sign, symptom or disease), experienced by a trial participant and which is temporarily associated with the study treatment (interventions or control) and is related to the affected shoulder or to the study interventions or control treatments.

AEs, which might be expected with this injury and its treatments include surgical site infection, dislocation/instability, haematoma, neurovascular injury including axillary nerve palsy, pain including complex regional pain syndrome, delayed wound healing and/or wound dehiscence, intraoperative fracture, acromial stress fracture, scapular notching, heterotopic ossification, implant loosening or failure and humeral bone loss [12, 15, 46]. Additionally, AEs associated with anaesthesia such as cerebrovascular events, cardiovascular events including venous thromboembolism, urinary retention and respiratory tract infection can also be expected in this patient group.

Serious AEs (SAEs) are defined as any untoward and unexpected medical occurrence that:

1. Results in death
2. Is life-threatening
3. Requires hospitalisation or prolongation of existing inpatients’ hospitalisation
4. Results in persistent or significant disability or incapacity
5. Is a congenital anomaly or birth defect
6. Any other important medical condition that, although not included in the above, may require medical or surgical intervention to prevent one of the outcomes listed

For the purposes of the PROFHER-2 Trial, the following are not considered a SAE but will be considered an adverse event:

- Complications of anaesthesia or surgery (e.g., wound complications, infection, damage to a nerve or blood vessel and thromboembolic events)
- Secondary operations for infection; dislocation or instability; malunion; non-union; peri-prosthetic fracture; or for symptoms related to the metalwork

Causality and expectedness of any SAEs will be confirmed by the Chief Investigator or another delegated surgeon co-investigator. SAEs that are deemed to be unexpected and related to the trial will be notified to the Research Ethics Committee (REC) and Sponsor.

All AEs will be reported to the TSC and DMC. Where repeated AEs of similar type are observed, these will be discussed with the DMC and will be onward reported should concerns be raised in relation to the type of event and/or frequency observed. All participants experiencing SAEs will continue to be followed up until the end of their participation in the trial. At the end of trial follow-up, all-cause mortality in study participants will be checked by authorised staff after securing the appropriate approvals using the electronically available NHS Summary Care Records or the National Care Records Service.

### Frequency and plans for auditing trial conduct {23}

Central monitoring of consent and participant eligibility will be completed for 100% of participants alongside routine validation of all data collected during data processing. Annual audit of site files will occur for each participating site. No onsite monitoring will be conducted in PROFHER-2 unless prompted by concerns regarding trial conduct or participant safety.

Regular trial management group, TSC, and DMC meetings will be held to review trial conduct.

### Plans for communicating important protocol amendments to relevant parties (e.g. trial participants, ethical committees) {25}

Any protocol amendments will be approved by the Sponsor (South Tees Hospitals NHS Foundation Trust) and the Funder (NIHR HTA) prior to submission to the REC and the Health Research Authority (HRA). Documentation will be provided to study sites for their local review and implementation as required.

### Dissemination plans {31a}

Results from this study will be submitted to relevant peer-reviewed journals, irrespective of the magnitude or direction of effect. A publications policy will be generated in advance to detail authorship, acknowledgements and review processes for the main publications.

An executive summary will be sent to the National Institute for Health and Care Excellence and other relevant bodies, including Integrated Care Boards, to facilitate adoption of study findings into clinical practice. We will work with the relevant National Clinical Director in the Department of Health to help ensure the findings of the trial are considered when implementing policy and we will work with the Specialist Advisory Committees to incorporate the findings into the training curriculum for clinicians who will undertake treatment for these fractures.

A plain language summary of the study report will be produced and made available to participants, members of our user group, and relevant patient-focused websites. The PROFHER-2 Patient Advisory Group will be encouraged to actively participate in the dissemination of the conclusions of this study to ensure these are easily accessible to patients.

## Discussion

The PROFHER-2 trial is designed to provide a robust answer to guide the treatment of patients aged 65 or over who sustain three and four proximal humeral fractures with regard to the clinical and cost-effectiveness of RSA vs HA, and surgery vs non-surgical treatment. As a result of the COVID-19 pandemic, recruitment to PROFHER-2 was paused between March 2020 and August 2020. In view of the challenges posed by subsequent recovery of services, resumption of recruitment to the trial was slower than anticipated. With approval from the funder, the overall study timeline was therefore extended, and the timeline for completion of trial recruitment was extended from May 2021 to June 2023. During trial conduct, it was apparent that a higher proportion of participants (35%) had an associated joint dislocation than anticipated at the start of the trial (10%). That led to a higher number of participants randomised to the surgical comparisons (RSA vs HA). Therefore, in order to maintain power for all planned comparisons, the allocation ratio was changed to 1:1:1 (RSA:HA:NS). Other key amendments to the protocol approved by the Research Ethics Committee include allowing remote follow-up of patients by sites where necessary, which was in response to the changes in clinical services imposed by the COVID-19 pandemic. The results of this trial should provide high-quality evidence to guide clinical practice in the use of arthroplasty for patients aged 65 years or over who sustain more complex (3- and 4-part) proximal humeral fractures.

## Trial status

Recruitment to the PROFHER-2 trial began on 28th June 2018, recruitment is anticipated to finish on 30th June 2023 — after an extension to the recruitment period was made. The current protocol is v1.3 (20.04.2022).

### Supplementary Information

**Additional file 1.** List of participating sites.

**Additional file 2.** 16-73-03_Rangan_Full_Outcome_Letter.pdf.

**Additional file 3.** REC approval 238346 15.5.18.pdf.

**Additional file 4.** HRA / HCRW approval IRAS 238346 .20180515.pdf.

**Additional file 5.** PROFHER-2 Consent Form - V1.0 12.03.18.pdf.

## Data Availability

This document constitutes the full protocol. Datasets and statistical code used in this study will be available from the York Trials Unit on reasonable request following the completion of the trial.

## Appendix 2

**Table:**

**Timepoint**	**Pre-randomisation/baseline**	**Randomisation**	**Treatment delivery**	**6-month post-randomisation**	**12-month post-randomisation**	**24-month post-randomisation**
**Enrolment:**						
Eligibility screen	X					
Informed consent	X					
Baseline questionnaire	X					
Allocation		X				
**Assessments**						
OSS	X			X	X	X
EQ-5D-5L	X			X	X	X
X-ray	X^*a*^		X^*b*^	X		
Visual analogue scale	X			X	X	X
PROMIS	x			X	X	X
Grip strength unaffected arm	X					
Range of movement				X		
Complications				X	X	X
Further procedures				X	X	X
Resource use				X	X	X
Adverse events				X	X	X

^a^Also post-reduction image if fracture dislocation reduced without general anaesthesia

^b^Post-operative X-ray for RSA and HA patients only

## Abbreviations

AE: Adverse event
BESS: British Elbow and Shoulder Society
CACE: Complier average causal affects
CRFs: Case report forms
DMC: Data Monitoring Committee
HA: Hemiarthroplasty
HEAP: Health economic analysis plan
MI: Multiple imputation
NICE: National Institute of Health and Care Excellence
NS: Non-surgical treatment
OSS: Oxford Shoulder Score
PHF: Proximal humeral fracture
PROMIS: Patient-Reported Outcomes Measurement Information System
PSS: Personal Social Services
QALY: Quality-adjusted life year
REC: Research Ethics Committee
RSA: Reverse shoulder arthroplasty
SAE: Serious adverse event
SWAT: Study within a trial
TSC: Trial Steering Committee
YTU: York Trials Unit
