# Supplementary material for: Reverse shoulder arthroplasty versus hemiarthroplasty versus non-surgical treatment for older adults with acute 3- or 4-part fractures of the proximal humerus: study protocol for a randomised controlled trial (PROFHER-2: PROximal Fracture of Humerus Evaluation by Randomisation – Trial Number 2)
Source: Trials. 2023 Apr 13;24:270. doi: 10.1186/s13063-023-07259-3 (PMC10098225; doi:10.1186/s13063-023-07259-3)
Supplement: Supplementary file 1 — Additional file 1. List of participating sites. [file 13063_2023_7259_MOESM1_ESM.docx]

**PROFHER 2: List of participating sites**

1. South Tees Hospitals NHS Foundation Trust
2. Oxford University Hospitals NHS Foundation Trust
3. Barts Health NHS Trust
4. Maidstone and Tunbridge Wells NHS Trust
5. North Bristol NHS Trust
6. James Paget University Hospitals NHS Foundation Trust
7. Great Western Hospitals NHS Foundation Trust
8. University Hospitals of North Midlands NHS Trust
9. United Lincolnshire Hospitals NHS Trust - Lincoln County
10. United Lincolnshire Hospitals NHS Trust - Pilgrim Hospital
11. Sherwood Forest Hospitals NHS Foundation Trust
12. Kings College Hospitals NHS Foundation Trust
13. Salisbury NHS Foundation Trust
14. Epsom and St Helier University Hospitals NHS Trust - SWLEOC
15. Cambridge University Hospitals NHS Foundation Trust
16. South Tyneside NHS Foundation Trust
17. Tameside and Glossop Integrated Care NHS Foundation Trust
18. Yeovil District Hospital NHS Foundation Trust
19. Country Durham and Darlington NHS Foundation Trust
20. North Tees and Hartlepool NHS Foundation Trust
21. Calderdale and Huddersfield NHS Foundation Trust Royal Infirmary
22. Nottingham University Hospitals NHS Trust
23. University Hospitals Of Leicester NHS Trust
24. University Hospitals Coventry and Warwickshire
25. NHS Grampian
26. Taunton and Somerset NHS Foundation Trust
27. Northern Devon Healthcare NHS Trust
28. Sandwell and West Birmingham Hospitals NHS Trust
29. Mid Yorkshire Hospitals NHS Trust
30. Northumbria Healthcare NHS Foundation Trust
31. NHS FIfe
32. The Queen Elizabeth Hospital Kings Lynn NHS Foundation Trust
33. Kingston Hospitals NHS Foundation Trust
34. Warrington and Halton Hospitals NHS Foundation Trust
35. Lewisham and Greenwich NHS Trust
36. Imperial College Healthcare NHS Trust
37. St George University of London and St George University Hospitals NHS Foundation Trust
38. Norfolk & Norwich University Hospitals NHS Foundation Trust
39. University Hospitals of Morcambe Bay NHS Foundation Trust
40. Milton Keynes University Hospital NHS Foundation Trust
41. Homerton University Hospitals NHS Trust
42. Hampshire Hospitals NHS Foundation Trust
43. Forth Valley Health Board
44. North Cumbria Integrated Care NHS Foundation Trust
45. NHS Greater Glasgow and Clyde
46. University Hospital Southampton NHS Foundation Trust
