# Supplementary material for: Reverse shoulder arthroplasty versus hemiarthroplasty versus non-surgical treatment for older adults with acute 3- or 4-part fractures of the proximal humerus: study protocol for a randomised controlled trial (PROFHER-2: PROximal Fracture of Humerus Evaluation by Randomisation – Trial Number 2)
Source: Trials. 2023 Apr 13;24:270. doi: 10.1186/s13063-023-07259-3 (PMC10098225; doi:10.1186/s13063-023-07259-3)
Supplement: Supplementary file 5 — Additional file 5. PROFHER-2 Consent Form - V1.0 12.03.18.pdf. [file 13063_2023_7259_MOESM5_ESM.pdf]

PARTICIPANT CONSENT FORM

PROFHER-2: ***PROximal Fracture of the Humerus: Evaluation by Randomisation Trial no. 2***

If you wish to take part in the PROFHER-2 study, **please place your initials in each of the boxes below, sign and date this form. <u>All the information on this form will be kept confidential and will not be released to anyone outside the research team</u>**

Please initial each box

1. I confirm that I have read and understand the information sheet version [*XX*] dated [*XX*] for the above study and have had the opportunity to ask any questions about the study and any questions have been answered to my satisfaction.	*Initials*
2. I understand that my participation is voluntary and that I am free to withdraw at any time without giving any reason, and without my medical care or legal rights being affected.	*Initials*
3. I agree to University of York's Trials Unit holding copies of my consent form, other study related documents and my contact details to allow them to send me questionnaires.	*Initials*
4. I agree to my GP being informed of my participation in the study and being advised of any significant information relating to my health that comes to light during my participation.	*Initials*
5. I understand that if I withdraw from the study, I will be asked to confirm that data collected from me can be used in analysing the results of the trial and that my personal details can continue to be stored. I will also be asked to confirm that my hospital records may be accessed for further outcome data collection. I understand that my identity will remain anonymous.	*Initials*
6. I understand that relevant sections of my hospital/GP medical notes and data collected during the study, may be looked at by individuals from University of York's Trials Unit, from regulatory authorities, the Sponsor or from the NHS Trust, where it is relevant to my taking part in the research. I give permission for these individuals to have access to my records.	*Initials*
7. I consent to the storage, including electronic, of personal information for the purposes of the study by [INSERT SITE NAME] NHS Hospital, the Sponsor and University of York Trials Unit. I understand that any information that could identify me will be kept strictly confidential and that no personal information will be included in the study report or other publication.	*Initials*
8. I understand that after this study has finished, the data collected from me will be securely stored in accordance with requirements of the law after which arrangements for confidential destruction will take place. Information that could identify me will be kept strictly confidential and no personal information will be included in the study report or other publication.	*Initials*
9. I understand that the information collected about me will be used to support other research in the future, and may be shared anonymously with other researchers	*Initials*
10. **I agree to take part in the PROFHER-2 Study.**	*Initials*

In addition to the above statements please initial the following box if you agree with the following statement. Your participation in this research study will not be affected if you do not agree with the statement.

11. I consent to the University of York's Trials Unit, regulatory authorities, the study Sponsor or the NHS Trust, accessing relevant information from the National Joint Registry, Hospital Episode Statistics and linked data sources to enable longer term follow up only in relation to my shoulder injury. I give permission for these individuals to have access to my records. Initials

Print name	d d / m m / y y y y	Signature
	__ __ / __ __ / 2 0 __ __	
Name of participant (*please print*)	Date	Signature of participant

Print name	d d / m m / y y y y	Signature
	__ __ / __ __ / 2 0 __ __	
Name of person taking consent (*please print*)	Date	Signature of person taking consent

1 for participant; 1 (original) for Investigator Site File; 1 for medical notes; 1 for York Trials Unit
