# Supplementary material for: Reverse shoulder arthroplasty versus hemiarthroplasty versus non-surgical treatment for older adults with acute 3- or 4-part fractures of the proximal humerus: study protocol for a randomised controlled trial (PROFHER-2: PROximal Fracture of Humerus Evaluation by Randomisation – Trial Number 2)
Source: Trials. 2023 Apr 13;24:270. doi: 10.1186/s13063-023-07259-3 (PMC10098225; doi:10.1186/s13063-023-07259-3)
Supplement: Supplementary file 2 — Additional file 2. 16-73-03_Rangan_Full_Outcome_Letter.pdf. [file 13063_2023_7259_MOESM2_ESM.pdf]

National Institute for Health Research

Evaluation, Trials and Studies Coordinating Centre
University of Southampton
Alpha House
Enterprise Road
Southampton
SO16 7NS

Tel: 023 8059 5586
Fax: 023 8059 5639
Email: htacommssioning@nihr.ac.uk

21 April 2017

Professor Amar Rangan
The James Cook University Hospital
Marton Road
Middlesbrough
North Yorkshire   TS4 3BW

Dear Professor Rangan

HTA Project: 16/73/03 - Effectiveness and cost-effectiveness of Reverse Shoulder Arthroplasty versus Hemiarthroplasty versus Non-surgical care for acute 3&4 part fractures of the proximal humerus in older adults - The PROFHER-2 Randomised Clinical Trial

Following the HTA Funding Board on 7 March 2017, and the budget review by the HTA Prioritisation Group, I am pleased to inform you that the HTA Programme has recommended funding for your above-titled project, subject to a satisfactory response to the Board's concerns, Finance and IP questions detailed below. Please would you inform your co-applicants of this decision.

Please note that this award is not secure until the work has been contracted, therefore, you must not publicise your achievement until you have received and signed a completed contract from us.

Please address the points raised below in a covering letter alongside an updated version of your full proposal and detailed project description.

Board feedback

- The board noted that applicants had responded positively to feedback raised at the expression of interest stage
- The applicants should clarify the exclusion criteria relating to all dislocations e.g. whether surgery would be necessary or not.
- The board would like reassurance that sufficient surgical expertise would be available to meet planned recruitment targets in each centre.
- The board would like to see reconsideration or further justification to explain why three separate statistical analyses had been chosen rather than analysis as a whole dataset with appropriate contrasts for the pairwise comparisons.
- The team should address how they will take account of variability in anaesthetic regimes and post-operative variations across sites
- The team should preplan the health economic analysis and pre-specify the progression criteria.
- The exclusion of individuals under 65yrs was not fully supported by the board and further justification needs to be provided.

- The HTA programme doesn’t fund training posts and the team should clarify the role of Mr Gwilym and any aspects relating to training should be removed.
- The board is aware of potential perceived conflicts of interest and would like to see further details how these will be managed in relation to funding support from the manufacturers.
- The team is asked to strengthen PPI by considering also involving patients with experience of non-surgical treatment.

Intellectual Property (IP) Feedback

- We would like to draw your attention to the draft contract, available at http://www.nihr.ac.uk/funding-and-support/funding-for-research-studies/manage-my-study/contracts-and-intellectual-property.htm and specifically to the background IP provisions. Please would you check what third party rights exist in terms of background IP. If there are none, please inform us, as this means there will be no requirement for schedule C in the contract. If third party rights do exist in relation to background IP, please provide details so that they may be appended to the contract in Schedule C.
- It is NIHR’s starting position that all arising foreground IP shall be held by the contractor. If you wish NIHR to consider alternative ownership arrangements, please provide details for consideration. The information you provide will feed into Schedule D of the contract.
- Applicants should provide reassurance that they have appropriate access rights to any data/ IP derived from the PROFHER study and to any other IP required to complete the study. Please provide the draft collaboration agreement(s) to support this.
- From the IP section ‘The other collaborators will be granted a royalty-free, non-exclusive license to use the new IP for teaching and research purposes’. This may be the best approach for the dissemination and utilisation of the foreground IP but the team should carefully consider all options and fully justify their approach.
- We encourage the team to consider options which will proactively influence the uptake of any conclusions from this study into clinical practice.

Finance comments on Research and NHS Costs

Our Finance Department has raised the following queries relating to figures in your application form that you need to respond to:

- Please check the salary calculations for Livio DiMascio and Amar Rangan as there appear to be some errors.
- Please confirm the number of planned international conference attendances over the duration of the project.
- General consumables and stationery should be covered by the indirect costs; please therefore either remove the York printing and stationery charge or explain what it covers.
- We are not able to cover NHS room hire, please remove this.
- Projects that register with the NIHR portfolio will not be charged for the ISRCTN; please therefore remove this.
- Please clarify the primary purpose of the x-rays that have been included as research costs.
- The 'usual treatment costs' need to be estimated and entered onto the application form. This should include the cost of treating 380 patients via the current standard care pathway outside of the trial by estimating the number of patients that would receive each treatment if not in the trial. With regard to the x-rays that have been included as NHS treatment costs, if they are carried out routinely as part of the standard care pathway they need to be re-entered as 'usual treatment costs' as well.

If you require any additional advice about the attribution of costs, please contact the Support Costs team, email: nets-finance@nihr.ac.uk. Clarification of these costs is essential and they will need to conform to the DH guidance on costs, known as AcoRD, see: https://www.gov.uk/government/publications/guidance-on-attributing-the-costs-of-health-and-social-care-research .

We do not wish to slow down your project start-up but all these points will need to be addressed to the satisfaction of all concerned. The result of any discussions should be outlined in the response letter you send.

The open comments supplied by the peer reviewers concerning your proposal were sent to you to allow you to respond to their comments in advance of the Board. These comments are available under 'view all my tasks' in the MIS and they may provide useful expert feedback as you prepare to begin your project.

Research Governance

HRA Approval is the process that covers research in the NHS in England. It replaces the need to make separate REC and R&D applications in England. HRA Approval comprises a review by a Research Ethics Committee and an assessment of regulatory compliance and realted matters undertaken by dedicated HRA staff. It is being introduced during 2015. Information for applicants is provided on the HRA website at http://www.hra.nhs.uk/resources/hra-approval-applicant-guidance/. Details on the assessment that studies will undergo and the standards that they will be required to meet to receive HRA Approval are available here: http://www.hra.nhs.uk/resources/hra-approval-applicant-guidance/hra-assessment-criteria-and-standards.

Sponsors and applicants are strongly encouraged to familiarise themselves with the latest version of these standards. Information on the responsibilities of sponsors is provided at http://www.hra.nhs.uk/resources/before-you-apply/roles-and-responsibilties/sponsor.

Project Start Date

The National Institute for Health Research (NIHR) is committed to timeliness of research and rapid initiation of studies following funding board assessment. As part of work to help reduce delays in project set-up, the NIHR, HRA and DH agreed a change to the standard contract permitting release of payments in advance of receipt of ethical approval (see website for details).

We are keen to see this project get started and completed for the benefit of patients as soon as possible and you should start to prepare your protocol and be working towards gaining the necessary governance approvals required for you to start the project within a maximum of 8 months of receipt of this letter.

As a programme, we wish to act in a fair and reasonable way and understand that there are often multiple reasons for delays and some factors that are outside of your control, however, we anticipate that the study will have started by at least 22 December 2017 and, as Chief Investigator for the study, you are responsible for ensuring the project starts in a timely fashion. We reserve the right to reconsider our offer of funding should you not start by this date.

Integrated Research Application System (IRAS) identification number

If your project will require ethical approval and/or any other governance approvals, please provide us with the IRAS identifier for this project in your letter of response to the Board, or justification of why it is not required for your project. Please note that you do not need to go

through the full process of submitting an ethics request to obtain this; recent changes on the IRAS website http://www.myresearchproject.org.uk/ mean that you are able to obtain an IRAS identifier simply by registering your study. Collecting the identifier at this stage is part of a pilot to try to smooth the permissions and approvals pathway for researchers as part of the Health Research Authority's work.

Next Steps

Please log into your user account for the NETSCC Management Information System (NETSCC MIS) at https://netscc-mis.nihr.ac.uk to respond to the comments and requirements for changes. You will have access to the relevant parts of your application form to revise and resubmit with the required changes.

Additionally, as part of the submission, you should include a letter that responds to each comment point in turn and clearly explains your proposed action(s). You should also provide a revised copy of your detailed project description which shows all the amendments as 'tracked changes'.

Both these documents should be included as **PDF** uploads when submitting your revised application (please note that you should delete any old versions currently showing on the MIS before uploading the revised PDF versions).

Please ensure that we receive your response to the above feedback by **1pm on Friday 19 May 2017.**

The post-award set-up process will be managed by Dawn Kean in the Monitoring team. Please direct all responses and future communications to her at **netspostawardsetup@nihr.ac.uk** or on 023 8059 7481.

Once the contract process is complete, the HTA Programme encourages successful applicants to consider submitting their protocols for review to journals such as the Lancet.

Yours sincerely

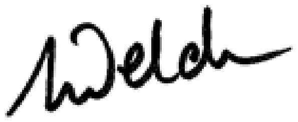

Linda Welch
Assistant Research Manager
E-mail: htacommissioning@nihr.ac.uk
Direct Tel: 02380 597527

NIHR HTA Programme responsibilities on behalf of the Department of Health

1. To assess the scientific quality of the research as proposed.
2. To establish the value for money of the research proposed.
3. To consider the suitability of the research environment, particularly the experience and expertise of the chief investigator and other key researchers.
4. To ensure the sponsor takes on all appropriate responsibilities before the research begins.
5. To comply and co-operate with any necessary enquiry, audit or investigation.
6. To ensure that the study has ethical and any other necessary approvals before funding is released.
7. To ensure the appropriate indemnities are in place in line with contractual arrangements.
8. To appoint trial management committee chairs and members and their terms of reference.
9. To authorise and approve payments in line with contractual arrangements.
10. To work with the chief investigator to publish information concerning the trial and its findings in the HTA monograph, in line with contractual arrangements.
11. To assess progress, in line with contractual arrangements.
12. To authorise and record changes to protocol, in line with contractual arrangements.
13. To hold site visits and project initiation meetings when appropriate, in line with contractual arrangements.
