# Supplementary material for: Reverse shoulder arthroplasty versus hemiarthroplasty versus non-surgical treatment for older adults with acute 3- or 4-part fractures of the proximal humerus: study protocol for a randomised controlled trial (PROFHER-2: PROximal Fracture of Humerus Evaluation by Randomisation – Trial Number 2)
Source: Trials. 2023 Apr 13;24:270. doi: 10.1186/s13063-023-07259-3 (PMC10098225; doi:10.1186/s13063-023-07259-3)
Supplement: Supplementary file 3 — Additional file 3. REC approval 238346 15.5.18.pdf. [file 13063_2023_7259_MOESM3_ESM.pdf]

Health Research Authority
North East – Tyne & Wear South Research Ethics Committee

HRA Newcastle
Newcastle Blood Donor Centre
Holland Drive
Newcastle upon Tyne
NE2 4NQ

Telephone: 0207 1048084

Please note: This is the favourable opinion of the REC only and does not allow you to start your study at NHS sites in England until you receive HRA Approval

15 May 2018

Professor Amar Rangan
Consultant Orthopaedic Surgeon
South Tees Hospitals NHS Foundation Trust
Department of Trauma and Orthopaedics
The James Cook University Hospital
Marton Road, Middlesbrough
TS4 3BW

Dear Professor Rangan

**Study title:**	**A three-arm randomised controlled trial to assess the effectiveness and cost-effectiveness of reverse shoulder arthroplasty versus hemiarthroplasty versus non-surgical care for acute three and four- part fractures of the proximal humerus in patients over 65 years of age – Proximal Fracture Of the Humerus: Evaluation by Randomisation trial No.2 (PROFHER-2).**
**REC reference:**	**18/NE/0125**
**IRAS project ID:**	**238346**

Thank you for your notification of 8 May 2018. I can confirm the REC has received the documents listed below and that these comply with the approval conditions detailed in our letter dated 2 May 2018

Documents received

The documents received were as follows:

Document	*Version*	*Date*
Participant information sheet (PIS) [Clean copy]	1.1	08 May 2018
Participant information sheet (PIS) [Tracked changes]	1.1	08 May 2018

Approved documents

The final list of approved documentation for the study is therefore as follows:

Document	*Version*	*Date*
Covering letter on headed paper [PROFHER 2 HRA Cover Letter]	1.0	15 March 2018
GP/consultant information sheets or letters [PROFHER 2 GP Letter]	1.0	12 March 2018
IRAS Application Form [IRAS_Form_16032018]		16 March 2018
Letter from funder [PROFHER 2 HTA Funding recommended letter]		08 June 2017
Letters of invitation to participant [PROFHER 2 CRF Cover Letter]	1.0	12 March 2018
Letters of invitation to participant [PROFHER 2 CRF Reminder Letter]	1.0	12 March 2018
Non-validated questionnaire [PROFHER 2 Participant CRF Mock Up]	1.0	12 March 2018
Other [PROFHER 2 Participant Contact Details Form]	1.0	12 March 2018
Other [PROFHER Expenses Claim Form - Patient Travel]	1.0	15 March 2018
Participant consent form [PROFHER 2 - Patient Informed Consent Form]	1.0	12 March 2018
Participant information sheet (PIS) [PROFHER 2 Sling Care Leaflet]	1.0	08 March 2018
Participant information sheet (PIS) [Clean copy]	1.1	08 May 2018
Participant information sheet (PIS) [Tracked changes]	1.1	08 May 2018
Research protocol or project proposal [PROFHER 2 Protocol]	1.0	09 March 2018
Sample diary card/patient card [PROFHER 2 Participant Visit Diary]	1.0	12 March 2018
Summary CV for Chief Investigator (CI) [A Rangan - CV 14.03.18]		14 March 2018
Validated questionnaire [Validated Questionnaire - Oxford Shoulder Score]		
Validated questionnaire [Validated Questionnaire - PROMIS Short Form]		
Validated questionnaire [Validated Questionnaire - EQ-5D-5L]		

You should ensure that the sponsor has a copy of the final documentation for the study. It is the sponsor's responsibility to ensure that the documentation is made available to R&D offices at all participating sites.

**18/NE/0125**	**Please quote this number on all correspondence**

Yours sincerely

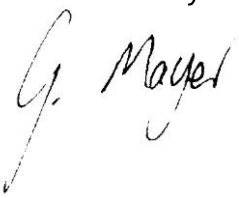

Gillian Mayer
REC Manager

E-mail: nrescommittee.northeast-tyneandwearsouth@nhs.net

Copy to: Mr Joe Millar – R&D Dept, South Tees Hospitals NHS Foundation Trust
