# Supplementary material for: Reverse shoulder arthroplasty versus hemiarthroplasty versus non-surgical treatment for older adults with acute 3- or 4-part fractures of the proximal humerus: study protocol for a randomised controlled trial (PROFHER-2: PROximal Fracture of Humerus Evaluation by Randomisation – Trial Number 2)
Source: Trials. 2023 Apr 13;24:270. doi: 10.1186/s13063-023-07259-3 (PMC10098225; doi:10.1186/s13063-023-07259-3)
Supplement: Supplementary file 4 — Additional file 4. HRA / HCRW approval IRAS 238346 .20180515.pdf. [file 13063_2023_7259_MOESM4_ESM.pdf]

Email: hra.approval@nhs.net
Research-permissions@wales.nhs.uk

Professor Amar Rangan
Consultant Orthopaedic Surgeon
South Tees Hospitals NHS Foundation Trust
Department of Trauma and Orthopaedics
The James Cook University Hospital
Marton Road, Middlesbrough
TS4 3BW

15 May 2018

Dear Professor Rangan

HRA and Health and Care Research Wales (HCRW) Approval Letter

**Study title:**	**A three-arm randomised controlled trial to assess the effectiveness and cost-effectiveness of reverse shoulder arthroplasty versus hemiarthroplasty versus non-surgical care for acute three and four- part fractures of the proximal humerus in patients over 65 years of age – Proximal Fracture Of the Humerus: Evaluation by Randomisation trial No.2 (PROFHER-2).**
**IRAS project ID:**	**238346**
**REC reference:**	**18/NE/0125**
**Sponsor**	**South Tees Hospitals NHS Foundation Trust**

I am pleased to confirm that **HRA and Health and Care Research Wales (HCRW) Approval** has been given for the above referenced study, on the basis described in the application form, protocol, supporting documentation and any clarifications received. You should not expect to receive anything further relating to this application.

How should I continue to work with participating NHS organisations in England and Wales?
You should now provide a copy of this letter to all participating NHS organisations in England and Wales*, as well as any documentation that has been updated as a result of the assessment.

*'In flight studies' which have already started an SSI (Site Specific Information) application for NHS organisations in Wales will continue to use this route. Until 10 June 2018, applications on either documentation will be accepted in Wales, but after this date all local information packs should be shared with NHS organisations in Wales using the Statement of Activities/Schedule of Events for non-commercial studies and template agreement/ Industry costing template for commercial studies.

Following the arranging of capacity and capability, participating NHS organisations should **formally confirm** their capacity and capability to undertake the study. How this will be confirmed is detailed in the "*summary of assessment*" section towards the end of this letter.

You should provide, if you have not already done so, detailed instructions to each organisation as to how you will notify them that research activities may commence at site following their confirmation of capacity and capability (e.g. provision by you of a 'green light' email, formal notification following a site initiation visit, activities may commence immediately following confirmation by participating organisation, etc.).

It is important that you involve both the research management function (e.g. R&D office) supporting each organisation and the local research team (where there is one) in setting up your study. Contact details of the research management function for each organisation can be accessed here.

How should I work with participating NHS/HSC organisations in Northern Ireland and Scotland?
HRA and HCRW Approval does not apply to NHS/HSC organisations within the devolved administrations of Northern Ireland and Scotland.

If you indicated in your IRAS form that you do have participating organisations in either of these devolved administrations, the final document set and the study wide governance report (including this letter) has been sent to the coordinating centre of each participating nation. You should work with the relevant national coordinating functions to ensure any nation specific checks are complete, and with each site so that they are able to give management permission for the study to begin.

Please see IRAS Help for information on working with NHS/HSC organisations in Northern Ireland and Scotland.

How should I work with participating non-NHS organisations?
HRA and HCRW Approval does not apply to non-NHS organisations. You should work with your non-NHS organisations to obtain local agreement in accordance with their procedures.

What are my notification responsibilities during the study?
The document "*After Ethical Review – guidance for sponsors and investigators*", issued with your REC favourable opinion, gives detailed guidance on reporting expectations for studies, including:

- Registration of research
- Notifying amendments
- Notifying the end of the study

The HRA website also provides guidance on these topics, and is updated in the light of changes in reporting expectations or procedures.

I am a participating NHS organisation in England or Wales. What should I do once I receive this letter?
You should work with the applicant and sponsor to complete any outstanding arrangements so you are able to confirm capacity and capability in line with the information provided in this letter.

The sponsor contact for this application is as follows:

Name: Joe Millar
Tel: 01642 854089
Email: stees.researchdevelopment@nhs.net

Who should I contact for further information?
Please do not hesitate to contact me for assistance with this application. My contact details are below.

Your IRAS project ID is **238346**. Please quote this on all correspondence.

Yours sincerely

Beverley Mashegede
Assessor

Email: hra.approval@nhs.net

Copy to: Mr Joe Millar, Sponsor Contact, Lead NHS R&D Contact

IRAS project ID	238346

List of Documents

The final document set assessed and approved by HRA and HCRW Approval is listed below.

Document	*Version*	*Date*
Contract/Study Agreement template [PROFHER 2 Unmodified mNCA]	1.0	14 March 2018
Covering letter on headed paper [PROFHER 2 HRA Cover Letter]	1.0	15 March 2018
GP/consultant information sheets or letters [PROFHER 2 GP Letter]	1.0	12 March 2018
IRAS Application Form [IRAS_Form_16032018]		16 March 2018
IRAS Application Form XML file [IRAS_Form_16032018]		16 March 2018
IRAS Checklist XML [Checklist_16032018]		16 March 2018
Letter from funder [PROFHER 2 HTA Funding recommended letter]		08 June 2017
Letters of invitation to participant [PROFHER 2 CRF Cover Letter]	1.0	12 March 2018
Letters of invitation to participant [PROFHER 2 CRF Reminder Letter]	1.0	12 March 2018
Non-validated questionnaire [PROFHER 2 Participant CRF Mock Up]	1.0	12 March 2018
Other [PROFHER 2 Participant Contact Details Form]	1.0	12 March 2018
Other [PROFHER Expenses Claim Form - Patient Travel]	1.0	15 March 2018
Participant consent form [PROFHER 2 - Patient Informed Consent Form]	1.0	12 March 2018
Participant information sheet (PIS) [PROFHER 2 Sling Care Leaflet]	1.0	08 March 2018
Participant information sheet (PIS) [Clean copy]	1.1	08 May 2018
Research protocol or project proposal [PROFHER 2 Protocol]	1.0	09 March 2018
Sample diary card/patient card [PROFHER 2 Participant Visit Diary]	1.0	12 March 2018
Summary CV for Chief Investigator (CI) [A Rangan - CV 14.03.18]		14 March 2018
Validated questionnaire [Validated Questionnaire - Oxford Shoulder Score]		
Validated questionnaire [Validated Questionnaire - PROMIS Short Form]		
Validated questionnaire [Validated Questionnaire - EQ-5D-5L]		

Summary of assessment

The following information provides assurance to you, the sponsor and the NHS in England and Wales that the study, as assessed for HRA and HCRW Approval, is compliant with relevant standards. It also provides information and clarification, where appropriate, to participating NHS organisations in England and Wales to assist in assessing, arranging and confirming capacity and capability.

Assessment criteria

Section	Assessment Criteria	Compliant with Standards	Comments
1.1	IRAS application completed correctly	Yes	No comments
2.1	Participant information/consent documents and consent process	Yes	No comments
3.1	Protocol assessment	Yes	No comments
4.1	Allocation of responsibilities and rights are agreed and documented	Yes	The Sponsor intends to use the unmodified mNCA 2018 version as the form of agreement with participating organisations.
4.2	Insurance/indemnity arrangements assessed	Yes	NHS Indemnity will apply.
4.3	Financial arrangements assessed	Yes	Funded by National Institute for Health Research.
5.1	Compliance with the Data Protection Act and data security issues assessed	Yes	No comments
5.2	CTIMPS – Arrangements for compliance with the Clinical Trials Regulations assessed	Not Applicable	No comments
5.3	Compliance with any applicable laws or regulations	Yes	No comments
6.1	NHS Research Ethics Committee favourable opinion received for applicable studies	Yes	Favourable Opinion with conditions issued 02 May 2018. Acknowledgement of conditions met issued 15 May 2018.

Section	Assessment Criteria	Compliant with Standards	Comments
6.2	CTIMPS – Clinical Trials Authorisation (CTA) letter received	Not Applicable	No comments
6.3	Devices – MHRA notice of no objection received	Not Applicable	No comments
6.4	Other regulatory approvals and authorisations received	Not Applicable	No comments

Participating NHS Organisations in England and Wales

This provides detail on the types of participating NHS organisations in the study and a statement as to whether the activities at all organisations are the same or different.
This is a non-commercial study and there was one site type.<br><br>The Chief Investigator or sponsor should share relevant study documents with participating NHS organisations in England and Wales in order to put arrangements in place to deliver the study. The documents should be sent to both the local study team, where applicable, and the office providing the research management function at the participating organisation. Where applicable, the local LCRN contact should also be copied into this correspondence.<br><br>If chief investigators, sponsors or principal investigators are asked to complete site level forms for participating NHS organisations in England and Wales which are not provided in IRAS, the HRA or HCRW websites, the chief investigator, sponsor or principal investigator should notify the HRA immediately at hra.approval@nhs.net or HCRW at Research-permissions@wales.nhs.uk. We will work with these organisations to achieve a consistent approach to information provision.

Principal Investigator Suitability

This confirms whether the sponsor position on whether a PI, LC or neither should be in place is correct for each type of participating NHS organisation in England and Wales, and the minimum expectations for education, training and experience that PIs should meet (where applicable).
A PI is expected at each participating organisation.<br><br>GCP training is not a generic training expectation, in line with the HRA/HCRW/MHRA statement on training expectations.

HR Good Practice Resource Pack Expectations

This confirms the HR Good Practice Resource Pack expectations for the study and the pre-engagement checks that should and should not be undertaken
All study activities will be undertaken by local staff employed by the NHS organisation. Therefore no honorary research contracts or letters of access are expected for this study.

Other Information to Aid Study Set-up

This details any other information that may be helpful to sponsors and participating NHS organisations in England and Wales to aid study set-up.
The applicant has indicated that they intend to apply for inclusion on the NIHR CRN Portfolio.
